# Pain, medicine and the monitoring of war violence: the case of rifle bullets (1868–1918)

**DOI:** 10.1017/mdh.2022.4

**Published:** 2022-04

**Authors:** Taline Garibian

**Affiliations:** Maison de l’histoire, University of Geneva, 5 rue de-Candolle, CH-1211, Genève 4, Switzerland

**Keywords:** Medicine, Warfare, Violence, Bullets, Wounds, Ballistics

## Abstract

The St Petersburg declaration, signed in 1868, is a milestone in the history of warfare and humanitarian law, as it prohibits the use of explosive bullets, which are considered to cause unnecessary suffering. As this article shows, the framing of this declaration that put suffering at its centre, as well as the development of the humanitarian movement, favoured the birth of a new field of expertise: wound ballistics. The wars that broke out after the declaration was signed are the subject of intense scrutiny, while the advances in weaponry, and notably, the creation by the British of a new expansive bullet, provided physicians with new fields of investigation. Numerous experiments have attempted to reproduce the effects of bullets on different materials, including corpses. Based on numerous medical reports and publications, as well as military archives from France and the United Kingdom, this investigation critically examines the notion of pain, its assessment and its use in the monitoring of war violence. It argues that, paradoxically, the greater attention paid to suffering has resulted in a need to objectify pain. This rationalisation and the quest for the quantification of suffering have not been without bias and have shifted attention away from care and treatment.

In July 1899, the *British Medical Journal* (*BMJ*) echoed the discussions held at the Hague conference concerning the use of weapons of war and, more specifically, explosive projectiles.^1^ The article, written by Alex Ogston, followed a series of pieces published a few months earlier in the same medical journal regarding the dumdum bullets – a recently developed British ammunition, designed to deform in the body and cause more severe injuries. As Western nations were in the process of establishing an international law of war, it is not surprising that discussions in relation to the lawfulness of certain munitions took place. However, more interesting is the role of medical expertise in this process. Indeed, the second half of the nineteenth century is characterised by the peculiar tendency of doctors to invest in the field of military regulation when the emergence of humanitarian practices and discourse allowed room for discussion regarding weapons, the nature of the injuries they caused and their legitimacy.

This article addresses how medicine contributed to the monitoring of war violence in the context of the nascent international law of war. By looking more specifically at the debates and experiments surrounding the use of explosible rifle bullets in France and Great Britain, I identify the process of objectification of harm that stems from medical practices, whether humanitarian or military. It is less a question of describing medical advances in healing and anti-pain techniques than considering the conditions necessary for the establishment of a level of expertise in terms of wounds’ assessment. Although the medical profession has been interested in war injuries for several centuries, the particular attention paid to the assessment and measurement of suffering from the second half of the eighteenth century onwards represents a significant evolution. In fact, even if neither the medical care of war wounds nor the study of the destructive power of bullets are new fields of study, they developed significantly during this period. Moreover, I would like to argue here that the emerging international law and humanitarian practices imposes a third issue, that of suffering or pain and its assessment. This contributed to modifying the chivalric ideal which dominated the western conflict culture and integrating a more rational stance on violence and battlefield behaviour among the military command at least.

The development of humanitarian interventions is closely linked to the management of war-wounded, since the First Geneva Convention, signed in 1864, is precisely dedicated to this issue.^2^ Moreover, the willingness to formally control the ability of small arms, intended to harm, dates back to the St Petersburg Declaration signed in 1868, well before the invention of the so-called dumdum bullet. The treaty hence prohibits ‘the employment of arms which uselessly aggravate the sufferings of disabled men or render their death inevitable’.^3^ Although limited in scope, this treaty is innovative in making suffering the criterion for banning certain weapons and prepared the ground for the intervention of physicians in the definition of thresholds of violence, even if they were not at the origin of this new approach. This rational reading of violence, based on suffering, that tended to impose itself in the elaboration of international law laid the foundations of forensic medicine as a humanitarian practice. Paradoxically, it also contributed to making the soldiers’ self-expression of pain less audible to physicians and left room for the biased interpretations of pain, based on the preconceptions of those who suffered from it.

Several historians have highlighted the subjectivity of pain and the tortuous paths that lead to its expression.^4^ The First World War provided historians with a significant number of sources on pain and suffering. The accounts of soldiers have proven to be particularly useful in capturing the expression of war suffering. In *The Politics of Wounds*, Ana Carden-Coyne relies on the voices of wounded servicemen to capture the experience of pain in its social, political and cultural dimensions.^5^ This work is an important contribution to the history of care and patients during the war and complements studies on military and humanitarian medicine.^6^ In the same vein, the corporeal dimension of war has attracted more attention and scholars have examined the experience of soldiers in war.^7^ More recently, scholars have also shed light on the conceptual shifts in the field of medicine triggered by the First World War.^8^ The case of the explosive bullets discussed here seeks less to account for the experiences of soldiers during the war than to understand how bodies became places of investigation. It is therefore situated at the intersection of the history of medicine, that of armaments and that of humanitarian practices and shows how they are intertwined.

## The birth of wound ballistics

The St Petersburg convention followed more than two decades of rapid technological advances in weaponry.^9^ For several years, explosive bullets were in use for hunts. In France, for example, the Devisme bullet was invented in 1857^10^ and was quickly used in the colonial empire during big game hunting parties.^11^ In 1863, the Russian army developed a projectile that exploded on contact with hard materials. It was aimed at destroying enemy cartridge cases and ammunition.^12^ This bullet was then enhanced to explode on contact with soft matter, such as human bodies.^13^ There were concerns that this weapon, intended to be used against enemy war material, was ultimately used by soldiers to target humans.^14^ This prompted states to legislate on their use in war. The convention, signed in St Petersburg, aimed to prohibit projectiles of a weight below 400g, which were either explosive or charged with fulminating or flammable substances. The agreement bound, notably, Austro-Hungary, Prussia, France, the United Kingdom, Italy, Russia and the Ottoman Empire, which renounced the use of these bullets during wars between themselves. The use of such ammunition against non-signatory parties or against the fighting population, as in the colonies, remained legal. The principles enshrined in the agreement signed in St Petersburg are a milestone in the history of humanitarian law, not only because their influence on current legal texts is noteworthy,^15^ but also because they frame the offence that puts pain at its centre.^16^

However, a few months after the St Petersburg convention, the Franco-Prussian War gave rise to allegations of the use of explosive bullets. Both sides accused one another of illicit violence. The Germans accused the French of various breaches of the laws of war, such as the unlawful execution of prisoners of war or the ill-treatment of care providers. In addition, the German documents state that prohibited bullets had been used by the French. Their accusation is detailed in a document in French, sent by Bismarck to Mac Mahon soon after the war.^17^ On the French side, the fact that the duchy of Baden had not ratified the St Petersburg convention became a cause of concern. Moreover, several caregiver accounts mentioned injuries consistent with the use of prohibited weapons.

After the war, the French national Amédée Tardieu, chief surgeon of the eighth ambulance of the *Société de secours aux blesses* [Wounded Relief Society], published a report on his activities between August 1870 and February 1871.^18^ In his medical observations, he mentioned the case of a soldier with gunshot wounds to both thighs. While the right thigh was penetrated by a bullet that left clear entrance and exit wounds and did not hit the femur, the left thigh appeared to have been more severely injured. Doctors noticed two clear holes that the injured man stated were the entry holes of the bullets and only one exit hole, which led them to conclude that a bullet remained in the body. Refusing at first to have his leg amputated, the soldier’s condition worsened and finally, he accepted the operation. This proved to be fruitless from a health point of view since the soldier died shortly afterwards, but it allowed doctors to perform a complete autopsy on the amputated limb. The physicians finally found many tiny pieces of lead, which had spread in the thigh, allowing them to conclude that an explosive bullet had been used.

These accounts, as accurate as they can be, were quickly challenged on both sides. In March 1871, the French doctor Nicaise published a text questioning the evidence used by the Germans to support their accusation. One of these pieces of evidence was the account made by Colonel Beckedorff in relation to his own wound, which occurred shortly after he witnessed small arms ammunition being fired nearby, causing a detonation. Nicaise explained that Beckedorff’s claim regarding the use of explosive bullets by the French relied solely on three elements. First, the pain the injury caused him, second, the piece of lead found in the body, away from the bullet trajectory and, finally, the black colour of this piece of lead. According to Nicaise, none of these elements were sufficient to prove the use of explosive bullets. Even if Nicaise admitted that ‘there is a lack of information on the nature of the pain produced by explosive bullets’,^19^ he considered that the pain expressed by Beckedorff would have been much more intense had it been an explosive bullet and that it was, therefore, a standard bullet. As for the presence of a piece of lead, the colour of which may have been due to the fact that it was in the body for a month, this does not correlate with the effect of explosive bullets, which tend to split into many pieces. Beyond the particular case of Beckedorff, this episode reveals the tension between the impressions of the wounded and their testimonies, the first observations of bodies and the debates that erupted in medical journals. As Joanna Bourke has shown, patients’ pain narratives from the eighteenth century were progressively sidelined by physicians, who no longer considered them as ‘contributing to accurate diagnosis’.^20^ Notable in the example mentioned above is the ambivalent position of the doctor, Nicaise, who, although conceded that the information on the pain caused by the explosive bullets was incomplete, also doubted Beckedorff’s testimony. In any case, although at this stage knowledge regarding pain was lacking, it could be a relevant factor in the evaluation of injuries.

A few months after Nicaise, another doctor, Thierry, examined the use of explosive projectiles by the Germans. First of all, Thierry commented on the weight given to accusations of violating the laws of war and the limited evidence on which they were based. ‘The newspapers of both sides echoed the reciprocal accusations with no supporting evidence other than the soldiers’ testimonies’.^21^ According to Thierry, certain physicians also had a tendency to take the injured at their word. Thierry, therefore, intended not only to deny the use of such projectiles, but to explain why so much credibility has been given to these allegations. The injured person used in the demonstration had suffered a major leg injury, characterised by the crushing of the femur by more than 15cm. According to Thierry, this injury can be explained by the fact that the bullet encountered an obstacle that cut it before it entered the body. This means that a ricochet injury can be quite damaging if the bullet maintains a high speed. Such a conclusion coincided with that of Dr Courtois, who emphasised the malleability and possible fragmentation of lead bullets. Overall, the controversy over the potential use of explosive bullets remained modest. Most doctors were sceptical of the veracity of these allegations, as reflected in the debate that took place in Metz at one particular conference, which involved the city’s doctors.^22^ The prevailing opinion was that no excessive use of explosive bullets could be attributed either to France or Germany, even if certain doctors did not exclude isolated cases. However, the injuries were subjected to careful attention, and many hypotheses were offered to explain the fragmentation of certain bullets; some doctors carried out experiments to test these.^23^ As Thierry revealed, the testimonies of the wounded alone were considered cautiously and medical practitioners tended to give weight to other elements.

The growing influence of medical expertise during the Franco-Prussian War contributed to the publicisation of war violence, even if, as Bertrand Taithe explained, ‘the war had not become any more brutal, yet brutality had become a focus of representations as the counterpoint of humanitarianism’.^24^ In the following decades, the debate on explosive bullets became very technical, from a ballistic and legal perspective. However, the dilemma involved not only the definition of a forbidden bullet and whether its use could be proven, but also the difficulty of determining when the use of highly damaging bullets was acceptable. Yet, as the notion of suffering is at the heart of the legislation, the measurement of the former became a medical issue as well as a political one.

## The dumdum bullet under scrutiny

The dumdum bullet originated in the struggle for colonial domination. It was intended in certain circumstances to replace the .303 inch Lee–Metford bullet, also known as Mark II, the efficiency of which was being questioned. Its appellation was coined from the name of the British arsenal location at Dum Dum in Bengal. The story of its development not only sheds light on conceptions of colonial warfare, but also demonstrates the involvement of medical science in the design of weapons. Military doctors examined wounds in the field, conducted experiments on animal cadavers and in this specific case, even benefitted from observing an execution.

In April 1895, Surgeon-Captain Hathaway was on duty when an execution was carried out. The executioners, six men from the First King Royal Rifle Corps, were armed with the Lee–Metford magazine rifle while the prisoner was tied. This gave Hathaway the opportunity to examine the effect of the bullets at short range. The wounds caused by the ammunitions were ‘well defined, with clean punched out edges [and] no injury to surrounding tissues’.^25^ The bones reached by the bullet appear to be drilled but not fractured. After examining the wound caused by a self-inflicted shot in the leg, the Surgeon-Captain concluded that ‘Although a very humane weapon, it is questionable whether it would stop a savage fanatical advance’.^26^ ‘Savage’, and ‘fanatics’, were common vocabulary in military literature concerning colonial opponents and were clear enough not to require further elaboration.^27^ This comment reveals what is at stake in these careful observations: did the weaponry need to be modified when used against certain populations?

In July 1895, medical officers serving under the command of the Chitral Relief Force were asked to provide reports on the nature of the wounds inflicted by the .303 inch Lee–Metford bullet.^28^ These reports were quite concerning for the military authorities. They concluded that someone struck by this bullet could actually walk ‘considerable distances for treatment’.^29^ More precisely, the entrance and exit wounds appeared to be similar and rather small, which meant that haemorrhaging was slow, the bullet did not break the bones and it did not cause shock or death.^30^ According to Surgeon-Colonel T. Maunsell, medical officer of the Chitral Relief Force, the bullet was likely to cause death when it reached vital parts of the body such as the brain, heart or great vessels, but was not very effective when it struck soft tissue.^31^ This view was supported by the findings of Surgeon-Lieutenant Jay Gould, made during the Chitral expedition. After examining and treating the injuries caused by the Lee–Metford bullet on the native population, he concluded that ‘The Lee–Metford is an excellent weapon in every respect but one, that is, would it stop a rush’?^32^ From 1895 onwards, the British army thus carried out experiments in order to produce ammunition that had a ‘greater stopping power’, so as to be used against ‘fanatical tribes’.^33^ According to a memorandum written by the Director-General of the Ordnance Factories:

It is quite possible to hit a man with any bullet in certain parts of his body, so that the result will not be knock down wound especially in the case of savage, who, when inflamed by fanatic, are notoriously unaffected by personal suffering, even of a most terrible nature.^34^

These observations jeopardise military dominance and, as Kim A. Wagner notes, ‘[it is] potentially extremely damaging to both British prestige and confidence in their own technological superiority’.^35^ Therefore, there can be a little doubt that the nature of colonial warfare prompted the British to consider new weapons. A bullet specifically designed for savage war was being considered.^36^ This initiative stemmed from the widespread opinion of a differing perception of pain according to race. ‘There is no doubt that Asiatics can stand wounds inflicted by sword or bullet infinitely better than Europeans can. Wounds that would kill a European, or at any rate lay him up for months, affect these hardy and abstemious mountaineers in a very much less severe manner’.^37^ Not only did it seem that local populations were more resistant to pain because of their ‘nervous development […] apparently less exalted than that of the European’, but their bodies also appeared to respond remarkably well to surgical treatment.^38^

The new type of soft-pointed ammunition was designed by Lieutenant Colonel Neville Sneyd Bertie Clay, superintendent of the Small Arms Ammunition Factory at Dum Dum. Several experiments were first carried out on two bullocks that were shot several times in different parts of their bodies. The superintendent also secured the assistance of a surgeon to examine bullock cadavers and reported the details of each of the injuries caused by the new bullets.^39^ The ammunition tested was a .303 inch bullet with the tip filed off, exposing the lead core and designed for setting up on impact. The modification was similar to that already patented by Major-General Tweedie.^40^ As reported in the *BMJ* in 1896, this new bullet was intended to enhance the Lee–Metford rifle, the ability of which to curb enemy assaults was deemed insufficient due to the small wounds it caused.^41^ However, concerns regarding the penetrative power of this new bullet were quickly expressed.^42^

The Indian context provided the British with opportunities to test their new ammunition, even if it was more difficult to observe the wounds inflicted on the enemies during fights. As the Director-General of the Ordnance Factories explains, ‘the only wounds examined and reported on are those on men who were not *killed*, but only *wounded* by the bullet, the Chitralis having apparently always carried off their dead’.^43^ Even if, according to the British army, the new bullet complied with the declaration of St Petersburg because it expanded in the body but did not explode, the ammunition was not intended to be used other than in the colonial context. Compliance with international agreements was as decisive as the nature of the conflict that made that type of bullet unnecessary. Military technologies and their use are shaped by their designers’ assumptions regarding enemies’ minds or feelings. ‘Savage warfare’ requires a specific bullet because of the cultural specificities attributed to the enemies, viewed as ‘fanatical tribes’. As Major General Walker phrased it in a letter to the Secretary to the Government of India: ‘I do not think, however, that a bullet which expands or becomes distorted on impact should be used in civilised warfare, nor would it be as necessary as when dealing with savage tribes ignorant of the possible effects of bullet wounds and indifferent to pain’.^44^ It is important to highlight that Major General Walker was more interested in the reaction to pain than the pain itself. According to him, the local population was not only insensitive to pain but also ignorant of the effect of their wounds, which made their behaviour irrational, unlike the British soldiers, who once wounded, waited patiently for help. Colonel La Garde stated 20 years later: ‘A fanatic like a Moro […] who never knows when he is hit until he is shot down must be hit with a projectile having a maximum amount of stopping power’.^45^ Thus, the physicians’ tools for measuring injuries were based on a distinctive understanding of the soldiers’ behaviour, namely that British or Western soldiers had a rational and measured attitude towards their wounds, an attitude that contrasted with the chivalrous ideal of the warrior fighting to the death. This differentiated understanding of fighting attitudes reveals the challenges of the quest for objectification of injuries. The reaction to pain, real or supposed, constituted a justification for a differentiated treatment of enemies, that signalled a departure from the universal code of honour that would govern combat. Here, pain was considered less of a measurable effect of an injury and more a physiological force, enhancing the strength of the sufferer and making him more dangerous. As the following shows, the desire to objectify war injuries conflicts with a rather unbalanced view of who should be protected by the laws of war.

## The science of wound production

The dumdum bullet soon became emblematic of a series of issues relating to the use of weapons and small arms ammunition in particular: first, because the designation of the dumdum bullet quickly and confusingly covered a broader range of small arms ammunition, ranging from expanding bullets (that deform in the body) to bullets loaded with explosible or inflammable substances. This made it more difficult to establish what was prohibited and what remained legal. Second, its context of origin raised the question of who was included in the notion of humanity promoted by the European powers. Basically, the dumdum bullet blurred the lines between legal and illegal weapons and human and inhuman use.

One might think that both issues are distinct; however, they have been intertwined from the very beginning, as General Hamilton’s argument showed. He attempted to clarify the misunderstanding that the dumdum bullet was not an explosive bullet since it was not loaded with powder. It was, therefore, authorised by international law, even if it expanded in the body.^46^ Yet, Hamilton was careful not to recommend its use in European wars. ‘As a rule when a ‘white man’ is wounded he has had enough, and is quite ready to drop out of the ranks and go to the rear; but the savage, like the tiger, is not so impressionable, and will go on fighting even when desperately wounded’.^47^ This argument reflects a form of embarrassment on the part of the British, as they refused to consider the dumdum bullet illegal but took great care nonetheless to reassure their European peers that it could potentially be used in Western conflicts.

Paul Von Bruns, a German professor of surgery based at Tubingen, who performed a series of trials with the new bullets, published his findings in a book before presenting them at the Hague Conference. Von Bruns also called the use of dumdum bullets ‘brutally inhumane’ in a meeting with the German Surgical Society in April 1898. This immediately prompted a response, published by Major Stevenson, reasserting the legality of this new ammunition in the columns of the *BMJ.* Stevenson’s argument was exemplary of the ambivalence of military doctors. It was a question of minimising the damage caused by these bullets while reaffirming their usefulness and, therefore, their effectiveness. According to the military surgeon, the effects of these bullets had been ‘exaggerated’. If the doctor was constrained to recognising the destructive power of the ammunition, the circumstances of the fighting justified its use. ‘That the dumdum bullet, when it breaks up, produces a more lacerated track through soft parts is not doubt true; for this it was invented, because the ‘stopping power’ of the Lee–Metford was found to be *nil* when bone was not implicated’.^48^ The ‘stopping power’ invoked here directly refers to a type of enemy and their presumed method of waging war. The dumdum bullets appeared to be more ‘effective against the rushes of Asiatic fanatics’.^49^ This indication was crucial. It served as a reminder that the use of dumdum bullets by the British army was a feature of colonial conflicts, to which the Convention of St Petersburg did not apply. However, Stevenson was keen to point out that, in his opinion, their use was not contrary to the St Petersburg Convention. Although the convention applied only between signatory countries, Stevenson wished to guard against possible moral recriminations of the same nature as that of Von Bruns: that is to say, the ‘inhumane’ nature of these bullets and not their potential illegality.

This article did not go unnoticed, as two British doctors, Alex Ogston, who was involved in several colonial campaigns, and Arthur E. Barker, published responses in the same journal. Both intended to downplay the discrepancies between Von Bruns and Stevenson. The debate that subsequently erupted in the journal revealed what was at stake in the dispute. First, according to Ogston, Von Bruns did not condemn the use of that projectile in general, and he was not strictly against the use of these bullets in a colonial context. As Ogston claimed, ‘Von Bruns expressly guarded himself from condemning the dumdum bullet in wars with barbarous races; his aim – a most laudable one in my estimation – was to arouse public opinion against the introduction into European warfare of bullets so far denuded of their mantle as to be virtually explosive’.^50^ Second, according to Ogston, the experiments conducted by Von Bruns involved a Moser-type bullet, the mantle of which was removed 1cm from the tip, rather than a bullet similar to the dumdum, the mantle of which was stripped only 2 or 3mm from the tip and was, therefore, less destructive.^51^

This debate thus went far beyond strictly medical issues. Medical expertise became a feature of the political debate in which blame was an important driver. As Joanna Bourke argued, ‘discourses of “humanity” (both as ontology and moral attitude) are at the heart of the ideology and practice of ballistics science’.^52^ Indeed, one of the questions that was at stake was whether these bullets should be used according to the humanitarian principles. The discussion initiated by Von Bruns led to several ballistic experiments that revealed the challenges posed by the principle of superfluous suffering and especially its evaluation. Experiments proved to be a key element in the medical understanding of war wounds and the comparison of bullets’ damaging power. Even though this kind of set up had been criticised, Arthur Keith and Hugh M. Rigby defended this method:

Objections have been made to the comparison of results obtained in the cadaver with those obtained in a living subject. The living body is practically a system of compartments filled and distended with fluid, and hence intensely susceptible to the well-known explosive effects. We obviated that error as much as possible by selecting a subject in which the amount of fluid in the tissues seemed to us to represent the amount present in life […].^53^

As sophisticated as they were, these experiments illustrated the ambivalence of war wound research, which on the one hand contributed to the development of the most effective weapon possible, and on the other, sought to determine whether the injuries were legally or morally acceptable. In both cases, there was a willingness to objectify war injuries. However, this twofold intention also revealed a form of paradox that has not been missed by Keith and Rigby, who took a negative view of the intervention of physicians in this debate. ‘Every weapon of war is “cruel, detestable and inhuman”, but whether the Dumdum bullet is unnecessarily so is rather a question for military experts than for medical men’.^54^ Thus, the attention paid not only to experimental procedures, but also to semantics and legal issues, clearly added another dimension to medical knowledge. While humanitarian principles have prompted debate on the use of certain bullets, the development of ballistic science was still strongly focused on what would later be termed ‘wound ballistics’,^55^ which was essentially a science of wound production.

## Wound ballistics and the monitoring of violence

The data produced by experimental devices fed into deliberations in The Hague. The views of the British delegation were clarified in a memorandum written by Sir J. Ardagh. Arguing that the use of dumdum bullets should remain lawful, even though they distort inside the body, he relied on the example of colonial wars. According to Ardagh, this was due to the attitudes of colonial fighters, whose body conceptions and responses to wounds differed from those of so-called civilised nations. In his memorandum, Ardagh, therefore, reaffirmed a position expressed orally: ‘your civilised soldier, […] when he has had a bullet through him, recognises the fact that he is wounded, and knows that the sooner he is attended the sooner will he recover’.^56^ In Ardagh’s mind, ‘civilised’ soldiers sought care as soon as they were hit by projectiles. On the contrary:

…[a] ‘fanatical barbarian, when he receives wounds of a like nature, which are insufficient to stop or disable him, continues to dash on, spear or sword in hand and before you have had time or opportunity to represent to him that his conduct is in flagrant violation of the understanding relative to the proper course for a wounded man to follow, he may have cut off your head’.^57^

The view of Ardagh thus echoed the aforementioned argument developed by Major General Walker, that westerners react more rationally when they are hurt. However, this opinion, imbued with the racial assumptions that led to the manufacture of these bullets, lacked support and Ardagh finally renounced the distinction between ‘savage’ and ‘civilised warfare’,^58^ even if privately, other delegates admitted that the distinction was relevant.^59^ It has been argued that Britain’s real intention was to use dumdum bullets indistinctively against all its opponents and that its focus on colonial warfare was only an ‘attempt to distract attention from its plans to use the ammunition against adversaries’.^60^ However, it is very likely that the British abandoned this position, which was no longer tenable in a final attempt to restore the image of the dumdum bullet, which they believed was indispensable in combating certain enemies. Indeed, several countries had spoken out against the distinction between civilised and savage opponents and had reaffirmed their desire to apply the same rules universally. They did not endorse the idea of a distinction between civilised war and savage war, which they regarded as irrelevant.^61^ In any case, on the ground, this distinction still appeared to be effective. This was evidenced by the opposition expressed by the military deployed on the field following the announcement of the discontinuation of the dumdum and Mark III bullets and the return to the Mark II bullet. In March 1901, for example, the officiating Adjutant General in India encouraged the Secretary of State to reconsider his decision regarding expanding bullets and reassured him: ‘Their use would therefore be only against savage tribes under no organised Government, who are not bound by any of the laws of civilised warfare’.^62^ This also prompted one to consider the discrepancies between the stance voiced publicly by the authorities and the ammunition used in practice, as well as the time needed for new binding rules to be accepted in all parts of the empire.

From the British perspective, the decisions taken in The Hague were definitely regarded as unjust. Ogston did not understand why the dumdum bullet was heavily criticised, especially since he claimed that false information was circulating regarding this ammunition.^63^ Moreover, he mocked the frank distinction made between ordinary gunshot wounds and dumdum gunshot wounds and castigated the idea that: ‘one who has a limb injured by a fully mantled bullet has before him but a few weeks of pleasant sojourn in bed, while the open-fronded bullets will cost him his limb by amputation, if not worse’.^64^ Hence, the signature of The Hague Convention did not put an end to the discussion on the use of violence; on the contrary, the concepts it relied on, such as ‘humanitarian’ or ‘unnecessary suffering’, gave new depth to the issue and confirmed the pre-eminence of medicine in this subject. This episode also revealed the limits of the law as, according to Joanna Bourke, the British ended up conforming to the prevailing view while ‘developing new projectiles as devastating as the dumdum but which stuck to the “letter but not the spirit” of the law’.^65^ The experts’ war begun by Ogston and Bruns over dumdum bullets foreshadowed the scrutiny that weapons would be subjected to in future conflicts. The multiple armed conflicts that occurred in the two decades preceding the First World War were as much an opportunity to observe the use of weapons on the field, as they were to measure the reactions they caused, particularly on the part of humanitarian organisations. As a result, conflicts that were sometimes very remote were the subject of close attention in Western Europe. That was, for example, the case as regards the Boer War, the Russo-Japanese War and the Balkan Wars.

It seems from the medical literature that expanding bullets were not widely used during the Boer War, although there may be certain exceptions. Arthur Keith and Hugh M. Rigby were in favour of this: ‘Nothing gave us greater satisfaction than to learn that neither our Mark IV nor our Dumdum bullet were to be used in South Africa’.^66^ This opinion was not only held by the British army doctors, but also by a German Red Cross mission, whose accounts were reported by Edmond Lardy in the Red Cross Journal. ‘The use of expanding bullets called Dumdum seems to have been the exception, but they were used, and Professor Küttner has brought back a complete collection of the various models of English Dumdum bullets’.^67^ As G. Fischer noted, the English were primarily using the Lee–Metford rifle, whereas the Boers used the Mauser and sometimes the Henry-Martini, the calibre of which was slightly bigger than the former two; the dumdum wounds still remained ‘excessively rare’.^68^ In general, doctors agreed that injuries inflicted during the South African War had a rather favourable prognosis and healed well. This led Clinton T. Dent to assert: ‘Broadly speaking, the injuries caused on the living subject are less serious than would have been anticipated from experiments. In this respect, then, the modern war is more humane than it was thought likely to be’.^69^

The favourable prognosis of gunshot wounds observed during the South African War, as well as in the Russo-Japanese War, led doctors to enthusiastically believe that the use of small-calibre bullets would make warfare more humanitarian. Some Western observers claimed that the efficiency of the Japanese military health services made the war more hygienic and humane.^70^ In France, the term *balle humanitaire* [humanitarian bullet] was even used to refer to those small calibre projectiles fully covered by a hard envelope. The paradoxes arising from a less-lethal bullet were immediately highlighted by some physicians. In 1893, an article published in the journal *L’Union médicale* pointed out the contradiction of the development of that kind of weapon:

Attempts had been made up to now to excuse the purveyors of death who, with all the power of their faculties stretched out towards this single object, are bent on discovering weapons, projectiles and explosives more surely homicidal, by asserting that these new devices would injure more only to kill less.^71^

However, by the beginning of the twentieth century, the benefits of small-calibre bullets with regard to wounds were universally acknowledged, although certain authors pointed out that since these bullets did not stay in the wound, they were likely to hit several targets in a row.^72^ This was also pointed out by the anonymous authors of *L’Union médicale*, who highlighted not only the consequence of that type of bullet on the healing process, but also the very nature of medical interventions in terms of multiplying the number of casualties.^73^ This raised the question of how violence control should operate; is it to limit the amount of damage that a single bullet can inflict, or is it to limit the amount of pain that can be inflicted on an enemy?

The decades leading up to the First World War were marked by an inflation of ballistic experiments. Numerous trials were carried out on various materials, including corpses and animals, to measure the effect of bullets. In 1908, for example, the British used thirteen sheep and seven horses to test the wounding power of the .303 inch bullets with pointed heads, compared to standard bullets.^74^ Most of the animals were killed before serving as targets, except for certain sheep, which were only chloroformed. The wounds were then assessed by means of a dissection carried out in a veterinary hospital. If these experiments focused on destructiveness, others tried to establish the correlation between damage and pain. Based on the strength of La Garde’s experiments on corpses, his observations during a visit to the military doctors at the Val de Grace in Paris and the reports of wounds inflicted in recent conflicts, he proposed a quantification of pain in 1914. ‘The amount of pain after the receipt of a gunshot wound depends upon the situation of the wound, its gravity and the amount of tissue involved in the traumatism’.^75^ According to this approach, pain is essentially determined by the physiological and biological characteristics of injuries and, therefore, seems to be easily measurable by a physician, even though the professor of military surgery acknowledged that a human response to pain is sometimes difficult to grasp. ‘Many of the men stated that they did not know when they were injured and they were ignorant of the presence of a wound until their attention was called to it by a comrade, or the sight of blood’.^76^ Indeed, as the numerous casualties of the First World War demonstrated, gunshot wounds and the pain they caused, were far from easy to assess.

## Assessing violence: the lessons of the First World War’s battlefields

Despite the international regulations that were put in place in St Petersburg and The Hague, the violence that characterised the fighting in the First World War was considerable, starting during the very first weeks of the conflict. Stéphane Audoin-Rouzeau and Annette Becker note that a tradition of self-containment of violence collapsed in the First World War.^77^ This is very true with regard to the Western world, but the colonial wars had already been the scene of extremely violent confrontation.^78^ Far from being confined to the military and politics, the violence of war raised questions among medical practitioners. As I will show, the scope of the convention of St Petersburg and subsequently that of The Hague regarding war projectiles, will be the subject of multiple debates, largely fuelled by observations made on the battlefield. During the first few months of the First World War, military medicine focused mainly on bullet wounds.^79^ These were causing the armies serious injuries, and the medical care of these wounds was proving to be more complicated than expected on the Western Front, due to the humid and muddy conditions which increased the risk of wound infection.^80^ This led the Surgeon-General, Sir Anthony Bowlby, to temper the medical and ballistic knowledge acquired so far. ‘In all old treatises on gunshot wounds, we find that the authors devoted their attention mainly to the nature of the projectile and its direct effects on the tissues of the body; but, important as are still these considerations at the present day, they must now be studied in conjunction with the terrain of war’.^81^ Indeed, the experimental conditions in which many bullets had been studied were not similar to the conditions on the war field. In this context, the prohibited projectiles were undoubtedly at the centre of attention, and many efforts to prove their use were expended.

A study of the French diplomatic archives shows that from the outset of war, military physicians on the field reported to their superiors’ instances of injuries which suggested an illegal use of weaponry. In August 1914, for example, two French doctors visited the bedside of a soldier wounded in Herbemont, Belgium. They stated:

Gunshot wound having penetrated the middle and outer part of the right forearm, has a very small diameter…The exit wound, located on the anterior side of the forearm, is a large crater of shredded flesh, twice the size of the palm of the hand. The radius is broken. And it is permissible to affirm under these conditions that this deep injury is due to the action of an explosive bullet; it is impossible that an ordinary bullet or any other projectile, after having produced such a clean entrance orifice, should cause such a large wound and such a bruising of the flesh at its exit.^82^

At the end of the report, the doctors added: ‘there can be no doubt in our minds, and our conviction is based on the comparison with the very many bullet wounds that we constantly have to treat’.^83^ The various reports that were produced pushed the military command to issue instructions.

The military government of Paris, after the observation made of a dumdum bullet wound at the Villemin Hospital and certified by Doctor Gaucher, invites the Director of the Health Service to have an urgent search made in all the hospitals of the entrenched camp for similar cases and to draw up a report relating to the place and date of the combat. Face and profile X-rays shall be taken as far as possible.^84^

X-ray images became a regular feature in the study of this type of gunshot injury. They were not only useful in the search for projectiles or projectile remains in the body, but they were also used to assess and demonstrate the extent of injuries caused by certain bullets. In 1914, the doctor Capitan delivered a presentation to the French Medical Academy, illustrated with an X-ray of wounds,^85^ whereas a few months later, doctor Lavielle published a paper on the use of dumdum bullets by the Germans on the western front in a French military journal, also providing X-rays and pictures of wounds.^86^ The increasing use of X-ray images certainly reveals the development of medical equipment in military and civilian hospitals. However, X-rays were also seen as a means of producing eloquent images of wounds that were easily disseminatable, although the technology used gave them a distinct medical aspect as opposed to photographs. X-rays thus provided a more detailed view of a wound and were regarded as a means to ‘by-pass patient-narrative in [the] search for an ‘objective diagnosis’.^87^ Clearly, the possible breaches of the laws of war were not the only reason for the use of objectification tools. The significant number of war wounded, and the social and financial stakes linked to pensions and compensation, were also considered. In addition to potential suspicions of malingering, physicians were concerned about soldiers exaggerating the extent of their injuries.

The projectiles themselves were also subject to a thorough examination. By ministerial order, the technical artillery section produced a report on ammunition used by the Germans in August 1914. The aim was to determine ‘(1) whether these projectiles can be considered as ‘dumdum’ bullets and (2) whether the use of such ammunition meets the conditions imposed by international agreements’.^88^

The reports produced by the French War Crimes Investigation Commission also looked into the use of prohibited ammunition in the volume dedicated to offences against soldiers and health care providers. Established in the decree of 23 September 1914, the purpose of this commission is to investigate acts committed in the regions most affected by the war. Composed of lawyers and senior officials, this commission aimed to draw up an inventory of the offences committed, town by town, by collecting the testimonies of civilians or military personnel. It compiled more than ten volumes, detailing all the offences reported during the invasion or the occupation, as well as offences against prisoners of war or civilians in captivity. These reports were widely publicised and had a huge impact.^89^ Regarding the question of the use of forbidden projectiles, the commission’s reports highlighted several different situations: projectiles turned over in the cartridge, cut, flattened or hollowed-out bullet tips or bullets with a longitudinally split envelope.^90^ Their work on small arms ammunition was based on ammunition found in the fields, on numerous testimonies and reports of soldiers, physicians or artillery specialists; however, there were few photographs of bullet wounds.

In September 1914, for example, the director of the technical section of the artillery, Lieutenant-Colonel Leleu, wrote a report on a German cartridge. He stated that the ammunition was of regulatory manufacture, but that its ogive – the pointed head of the bullet – was subsequently cross-incised. According to Leleu, this was not exactly a dumdum bullet but rather an artisanal model. ‘It is, as were the first English cartridges used in the Chitral, a do-it-yourself after-manufacture, operated nevertheless by a worker with a vice and a hacksaw. The operation therefore had to be carried out systematically on a certain quantity of ammunition’.^91^ The forensic pathologist, Dr Mesnard, chief surgeon of the Hospital of Saint Julien, also made observations at the request of a public prosecutor. He investigated several suspicious cases. Among them was the wound of a 24-year-old soldier named Hinterlang, who was shot in the arm during the battle of Ypres on 12 November 1914. The entrance wound was small and clear cut, whereas the exit wound was particularly significant. Mesnard took some photographs of the wounds, which he attached to his report. He concluded:

It seems difficult to me to admit that a projectile such as the German bullet could, with such a narrow entrance hole, produce such damage on its exit. I have not found any trace of the projectile, but it seems obvious to me that it is, according to the examination of the wound, a wound made by an explosive bullet, or at least a bullet with a cut or overturned tip.^92^

## Objectifying the harm

The issue of the use of forbidden projectiles was taken very seriously by leaders and soon became grounds for a war of information between European countries. In September 1914, the *Norddeutsche Allgemeine Zeitung* disclosed a telegram sent by Kaiser Wilhelm to President Wilson. The letter stated that forbidden ammunitions were found by German soldiers after having taken a French position.^93^ Other documents mentioned the use of explosive bullets by the British army, causing the War Office to issue a memorandum on this matter refuting German accusations.^94^

These early accounts probably prompted Stevenson to reiterate the modalities of use of these bullets, created to counter assaults during colonial conflicts; they should, according to him, not be used between ‘civilised nations’. However, he thought it unlikely that so-called civilised nations would use them against one other ‘to any great extent’, unveiling an idea that was likely to be widespread. Dumdum bullets were created for colonial warfare and were still intended for this type of conflict. His article denounced ‘young civilian surgeons’ on both sides who were inexperienced in dealing with bullet wounds and consequently accused their enemies of using dumdum or explosive bullets. Moreover, Stevenson insisted on the conditions under which the use of such weapons could be established with certainty. ‘The certain evidence of their employment is the finding of the bullets or this kind of fragment of them in the patients during their surgical treatment or in the position recently evacuated by the enemy’.^95^ He believed that wounds alone were not enough to prove the use of this ammunition, as they differed according to the size of the bullet, its velocity, the distance from which it was fired and the part of the body it hit (soft part, bones etc.) This position was backed by the German Professor, Karske, whose observations on the eastern front were reported in the *BMJ* a couple of weeks later.

Most of the wounds were due to rifle bullets, and in respect of shape, size, and degree there was no difference between the wounds of the Germans and the French. Most of the wounds had been inflicted at a range of 400 to 600 metres, and there was, therefore seldom any sign of so-called ‘explosive’ effects of the bullets.^96^

Karske’s piece came to the English audience along with several other translations of German studies on dumdum bullets, all published in the first instance in the *Münchner Medizinische Wochenschrift.* The claim that the wounds do not provide sufficient proof to assess the use of explosive devices did not put an end to the controversy that attracted many doctors’ attention. As before the war, the significant injuries caused by certain munitions raised doubts as to their compliance with the law. However, once again, the law was not the only standard to which physicians referred.

In a piece of work translated for the *Journal of the Royal Army Medical Corps*, the German surgeon, Stargardt, considered the effect of the British ammunition regardless of its accordance with international agreement. Obviously, this ammunition was his focus when he asked ‘whether, and to what extent, the Dumdum bullet differs from the regular English infantry bullet; and further, whether and to what extent the wounds inflicted by the Dumdum bullets differ from those inflicted by the regular English infantry bullet’.^97^ The scrutiny with which the British bullet was examined, stemmed from its destructive power directly observed by Stargardt. During the first 2 months of the war, German casualties were caused by the French, whose bullets were not very destructive, whereas, during the following month, when fighting against the British, the German troops suffered from more serious injuries. Stargardt’s argument was demonstrated by an X-ray examination of several wounds, caused by the British rifle. His conclusion was clear: ‘the usual English infantry bullet, without needing special treatment, causes wounds which are exactly like those caused by the Dumdum, and must be like them on the account of special construction’.^98^ This opinion was later contradicted by Kirschener, himself criticised by G. Perthes.^99^ However, the British army conducted experiments with Austrian bullets. Dead sheep and horse corpses were used to measure the destructive effect of this ammunition. Interesting in this case is the method used by the army to make their results conspicuous. After a post-mortem examination, the bones were displayed to make the effects of the explosion visible before being photographed.^100^ During the same period, gelatine was used in the United States in experiments to represent body material and simulate the effects of explosive devices on human tissue.^101^ Although the experimentation was increasingly more sophisticated, it was not possible to assess the injuries with certainty and to put an end to the controversies. As Cornish notes: ‘most reports of wounds allegedly caused by “dumdums” can be attributed to the effects of normal bullets, which “tumbled” after impact. In British tests on animal carcasses with Mk VII ammunition, carried out in 1911, no less than 63% of the bullets fired had behaved in this way’.^102^ That is to say, in many cases, the assessment of breaches of the laws of war is very difficult. Not only could regular bullets be modified by soldiers, but their trajectory and reaction could be altered before they hit their target. However, armies were increasingly considering the public impact of these reports. As a science of breaches of the laws of war, ballistic action could also be integrated into humanitarian action.

## Ballistic and humanitarian action

One of the most thorough investigations on forbidden ammunition during the war was certainly that conducted by Rodolph A. Reiss in Serbia. Reiss was a criminology professor at the University of Lausanne in Switzerland. Born in 1875 in Germany, he was trained in chemistry and was also an expert in forensic photography. In 1900, he went to Paris to complete his training with Professor Alphonse Bertillon, in charge of the laboratory of anthropometry at the Prefecture de Police.^103^ In 1906, Reiss was appointed professor of forensic science and, 3 years later, became the head of the Institute of Forensic Science in Lausanne. When, in 1914, he was commissioned by the Serbian government to investigate the atrocities committed by the invading Central Powers, he enjoyed a certain notoriety and an extensive network. His first observations were published in the French journal devoted to criminology: *Archives de l’anthropologie criminelle.* The analysis of certain bullets used by the Austrians in Serbia revealed the existence of a second firing pin inside the projectiles. This was triggered when the bullet encountered an obstacle and caused an explosion. This led Reiss to state: ‘This bullet therefore clearly presents all the characteristics of an explosive bullet as used until now for hunting pachyderms only’.^104^ Reiss did not only consider the small arms ammunitions, but also examined the wounds suffered by Serbian soldiers and fired test shots on a wooden board; he also questioned Austrian soldiers taken as prisoners. Moreover, the International Committee of the Red Cross was directly approached. In 1916, it commissioned a Swiss doctor to examine three bullet samples apparently used by Austrian soldiers. The samples were sent to the Red Cross Committee by a commander of the Italian army, together with a report produced by a doctor and photographs. This demonstrated not only a willingness to ensure that the breaches of the laws of war were recorded, but also a concern for objectivity. According to the Swiss doctor who examined this material, the second bullet was of particular interest; it was a standard bullet, the end of which had been cut laterally, in order to become deformed when penetrating the body.^105^ Even if this investigation did not progress further, these examples show that humanitarian organisations considered the use of ballistics and their political influence.

## Conclusion

In a remarkable book on wound ballistics published in 1962, E. Newton Harvey and his co-authors stated, ‘the body is heterogeneous and opaque’.^106^ The authors regretted that the wounds had been overlooked by ballistic specialists to date ‘in part, owing to the rapidity of changes which take place in an opaque medium and the difficulty of measuring them, and, in part, to the complexity of the body and the feeling that few significant generalisations could be made regarding it’.^107^ This tendency to downplay the work of those who came before them certainly stemmed from a desire to write the history of the discipline, starting with their own work. Although few doctors spoke with one voice and some were reluctant to base their work solely on wounds, many experiments and observations, sometimes using sophisticated technology, were made from the mid-nineteenth century onwards to assess the harm caused by bullets. Their results were often discordant. Although physicians were ingenious in creating experimental devices to evaluate projectiles, they were less rigorous in determining what was ‘humane’ and what was not, and to whom humanitarian principles should apply. This certainly made it more difficult for those who wished to limit universally the impact of injuries caused by small arms ammunition. However, the use of expanding or exploding bullets, as well as other ‘inhuman appliance[s]’ were among those included in the list of offences against the German Empire and its allies, drawn up at the peace conference following the First World War.^108^ However, the prosecution recorded poor outcomes and the conference made little mention of the offences committed against non-European populations.

The work of doctors on war injuries nevertheless reveals the quest for the objectification of pain, which, as shown, was not only driven by military objectives, but also by new humanitarian principles. Their work focused on measuring the efficiency of bullets, ie., their destructive effect and the pain they caused. Ballistics proved to be a science of wound production which implied assessments on the field during wars and the setting up of many experimental observations. This work served both the production of more effective weapons and the ascertainment of violations of the laws of war, by establishing a causal link between the use of a certain type of projectile and a given injury. This last task undoubtedly gave a humanitarian dimension to ballistic science. The infatuation aroused at the end of the nineteenth century by the small calibre bullets that the French called ‘humanitarian bullets’ was only of short duration, as the conflicts that followed proved to be devastating. Although pain and destructiveness were at the focus of physician’s attention, their approach left a little room for the subjective expression of pain. Indeed, the development of medical techniques for assessing injuries is coupled with a greater reluctance on the part of physicians to rely on the testimony of the injured. Moreover, many experiments proved that in certain circumstances, authorised bullets could be just as destructive and painful as prohibited ones. This tendency to focus on the weapon rather than the characteristics of the injury that has dominated the ballistic for over a century has only recently been nuanced by the classification of war wounds, developed by the Red Cross in the early 1990s. This text intends to fulfil a need for ‘a clinical classification of wounds that is based on the features of the wound and not on the weaponry or the presumed velocity of the missile’.^109^ Such a vision contrasts with the efforts described here, so as to establish a clear causal link between bullets, physiological damage and pain.

## Footnotes

1

Alex Ogston , ‘The Peace Conference and the Dum-Dum Bullet’, BMJ, 2, 2013 (1899), 278–81.

2

Tanisha M. Fazal , Wars of Law: Unintended Consequences in the Regulation of Armed Conflict (Ithaca; London: Cornell University Press, 2018), 15 .

3 Declaration Renouncing the Use, in Time of War, of Explosive Projectiles Under 400 Grammes Weight, Saint Petersburg, 29 November–11 December 1868. In its present wording, the rule is known as the prohibition of unnecessary suffering or superfluous injury.

4 See, for example, Elaine Scarry, ‘Injury and the Structure of War’, *Representations*, 10 (1985), 1–51; Joanna Bourke, ‘Bodily Pain, Combat, and the Politics of Memoires: Between the American Civil War and the War in Vietman’, *Histoire sociale/Social History*, 46, 91 (2013), 43–61.

5 Ana Carden-Coyne, *The Politics of Wounds, Military Patients and Medical Power in the First World War* (Oxford: Oxford University Press, 2014).

6 See, for example, Mark Harrison , The Medical War: British Military Medicine in the First World War (Oxford: Oxford University Press, 2010); Vincent Viet, *La santé en guerre, 1914–1918: Une politique pionnière en univers incertain* (Paris: Presses de Sciences Po, 2015).

7 Paul Cornish and Nicholas Saunders (eds), *Bodies in Conflict: Corporeality, Materiality, and Transformation* (London: Routledge, 2014).

8 Stefanos Geroulanos and Todd Meyers, *The Human Body in the Age of Catastrophe. Brittleness, Integration, Science, and the Great War* (Chicago: Chicago University Press, 2018).

9

Scott Keefer , ‘“Explosive Missals”: International Law, Technology, and Security in Nineteenth-Century Disarmament Conferences’, *War in History*, 21, 4 (2014), 445–64: 450.

10
*Encyclopédie de famille: répertoire général des connaissances usuelles* (Paris: Librairie de Firmin Didot frères, fils et c^ie^, 2, 1868), 282.

11 Ironically, this bullet was a ‘humanitarian’ innovation in the eyes of some commentators of the time, since it made it possible to hunt dangerous animals with less risk for the hunter; see ‘chronique scientifique’, *Revue Britannique,* 1862, 499.

12 ‘Annexe au Protocole I des Conférences militaires tenues à Saint-Pétersbourg, Mémoire sur la suppression de l’emploi des balles explosives en temps de guerre’, *Nouveau Recueil général de Traités et Autres Actes relatifs aux Rapports de Droit international*, XVIII (Göttingen, 1873), 458.

13

Emily Crawford , ‘The Enduring Legacy of the St Petersburg Declaration: Distinction, Military Necessity, and the Prohibition of Causing Unnecessary Suffering and Superfluous Injury in IHL’, Journal of the History of International Law, 20, 4 (2018), 544–66; 548.

14 Annexe, *op. cit.* (note 12), 460.

15 Crawford, *op. cit.* (note 13), 544–66.

16 Henri Meyrowitz remarked cogently that the notion of superfluous deaths has received a little attention. See Henri Meyrowitz, ‘Le principe des maux superflus: De la Déclaration de Saint-Pétersbourg de 1868 au Protocole additionnel I de 1977’*, International Review of the Red Cross*, 76, 806 (1994), 107–30.

17
*Les Violations de la Convention de Genève par les Français en 1870–1871. Dépêches, protocoles, rapports etc.* (Berlin: Éditeur Charles Duncker, 1871).

18 Amédée Tardieu, *8^em^ ambulance de campagne de la Société de secours aux blessés (campagnes de Sedan et de Paris), août 1870 – février 1871, Rapport historique, médical et administratif* (Paris: Adrien Delahaye libraire-éditeur, 1872).

19 Nicaise, ‘A propos des Balles explosives’, *La Gazette médicale de Paris*, 26, 11 (1871), 102–4: 103.

20

Joanna Bourke , The Story of Pain. From Prayer to Painkillers (Oxford: Oxford University Press, 2014), 132.

21 E. Thierry, ‘Des Balles explosibles’, *Gazette des hôpitaux civils et militaires*, 44, 153 (1871), 610.

22 E. Grellois, *Histoire médicale du blocus de Metz* (Paris and Metz: Librairie J.-B. Baillère et fils and Librairie M. Alcan, 1872), 358–9.

23 Jules Eugène Rochard, *Histoire de la chirurgie française au XIX^e^ siècle: Étude historique et critique sur les progrès faits en chirurgie et dans les sciences qui s’y rapportent depuis la suppression de l’académie royale de chirurgie jusqu’à l’époque actuelle* (Paris: Librairie J.-B. Baillière et fils, 1875), 855.

24 Bertrand Taithe, *Defeated Flesh: Welfare, Warfare and the Making of Modern France* (Manchester: University Press, 1999), 164.

25 H. Hathaway, ‘Notes on the effect on the human body of the Lee–Metford magazine rifle at a short range’, British Library, IOR/L/MIL/17/13/64.

26
*Ibid.*

27

Kim A Wagner , ‘Savage Warfare: Violence and the Rule of Colonial Difference in Early British Counterinsurgency’, History Workshop Journal, 85 (2018), 224.

28 Letter from Major General Morton to the Secretary to the Government in of India, 24 July 1895, British Library, IOR/L/MIL/17/13/64.

29
*Ibid.*

30
*Ibid.*

31 Letter from Surgeon Colonel T. Maunsell to the Chief Staff Officer of Chitral Relief Force, 19 June 1895, British Library, IOR/L/MIL/17/13/64.

32 Jay Gould, ‘On the Action of the Lee–Metford Bullet on Bone and Soft Tissues in the Human Body, Made During the Chitral Expedition’, *BMJ*, 2, 1803 (1895), 129–30: 130.

33 Letter from Maj. Genl. A. Walkers to the Secretary to the Government of India, 2 December 1895, British Library, IOR/L/MIL/7/12013.

34 Memorandum from the Director-General of Ordnance Factories, 13 August 1895, British Library, IOR/L/MIL/7/12013.

35 Wagner, *op. cit.* (note 27).

36 Minute by the Director General of Ordnance Factories, 29 July 1895. British Library, IOR/L/MIL/7/12013.

37 George John and Francis Edward Younghusband, *The Relief of Chitral* (London: MacMillan, 1910 [1895]), 90.

38 ‘Little Wars and the Small-Bore Rifle’, *BMJ*, 2, 1803 (1895), 153–4.

39 Surgical description of wounds caused by the service 303 inch bullet with the top filed off, exposing lead core, British Library, IOR/L/MIL/7/12013.

40

Edward M. Spiers , ‘The Use of the Dum Dum Bullet in Colonial Warfare’, The Journal of Imperial and Commonwealth History, 4, 1 (1975), 3–14; 4.

41 ‘The Military Bullet’, *BMJ*, 2, 1877 (1896), 810.

42 Minute by the Director General of Ordnance Factories dated 29 July 1895, British Library, IOR/L/MIL/7/12013.

43 Memorandum from the Director-General of Ordnance Factories dated 13 August 1895, British Library, IOR/L/MIL/7/12013.

44 Letter from Maj. Genl. A. Walkers to the Secretary to the Government of India, 2 December 1895, British Library, IOR/L/MIL/7/12013 Small arms.

45 Colonel Louis Anatole La Garde, *Gunshot Injuries: How They Are Inflicted, Their Complications, and Treatment* (New York: William Wood and Co., 1914), 66.

46 J.B. Hamilton, ‘The Evolution of the Dum-Dum Bullet’, *BMJ*, 1, 1950 (1898), 1250–1. Here, Hamilton wrongly mentions the Geneva declaration which does not refer to the use of small arms ammunitions.

47
*Ibid.*, 1251.

48 W.F. Stevenson, ‘The Effect of the Dum-Dum Bullet from a Surgical Point of View’, *BMJ*, 1, 1951 (1898), 1324–5.

49
*Ibid.*

50 Alex Ogston, ‘The Effects of the Dum-Dum Bullet from a Surgical Point of View’, *BMJ*, 1, 1953 (1898), 1490.

51 In fact, Von Bruns seems indeed to have use soft-nose Mauser bullet; see Alex Ogston, ‘The Wounds Produced by Modern Small-Bore Bullets. The Dum-Dum Bullet and the Soft-Nosed Mauser’, *BMJ*, 2, 1968 (1898), 813–15.

52 Joanna Bourke, ‘Theorizing Ballistics: Ethics, Emotions, and Weapon-Scientists’, *History and Theory*, 56, 4 (2017), 138.

53 Arthur Keith and Hugh M. Rigby, ‘Modern Military Bullets: A Study of Their Destructive Effects’, *The Lancet*, 154, 3979 (1899), 1499–507: 1499.

54
*Ibid.*

55 James C. Beyer (ed.), *Wound Ballistics* (Washington DC: Office of the Surgeon General Department of the Army, 1962).

56 Memorandum by Sir J. Ardagh respecting Expanding Bullets, The Hague, 14 June 1899, British Library, IOR/L/MIL/7/12028.

57
*Ibid.*

58
*Ibid.*

59 Keefer, *op. cit.* (note 10), 460.

60 Huw Bennett *et al.*, ‘Studying Mars and Clio: Or How Not to Write About the Ethics of Military Conduct and Military History’, *History Workshop Journal*, 88 (2019), 274–80: 275.

61 Memorandum by Sir J. Ardagh respecting Expanding Bullets, The Hague, 14 June 1899, British Library, IOR/L/MIL/7/12028.

62 Enclosure from the Officiating Adjutant-General in India, to the Secretary of the Government of India, 13 June 1901, British Library, IOR/L/MIL/7/12030.

63 Ogston, *op. cit.* (note 1), 279–80.

64
*Ibid.*, 279.

65

Joanna Bourke , Wounding the World. How Military Violence and War-Play Invade Our Lives (London: Virago Press, 2014), 73.

66 Arthur Keith and Hugh M. Rigby, ‘Modern Military Bullets: A Study of Their Destructive Effects’, *The Lancet*, 154, 3979 (1899), 1499–507: 1499.

67 Edmond Lardy, ‘Rapports des médecins allemands envoyé au sud de l’Afrique’, *Bulletin International des Sociétés de la Croix-Rouge*, 32, 126 (1901), 81–92: 83.

68 G. Fisher, ‘Les opération de la Croix-Rouge Allemande chez les Boers dans l’Afrique du Sud. (Contribution à l’étude de la chirurgie de guerre dans la campagne Sud-africaine)’, *Archives de médecine et de pharmacie militaires*, 37 (1901), 249–64: 253.

69 Clinton T. Dent, ‘A Lecture on Small-Bore Rifle Bullet Wounds and the “Humanity” of the Present War’, *BMJ*, 1, 2055 (1900), 1209–13: 1209.

70 Ken Daimaru, ‘Entre blessures de guerre et guerre des blessures: la “balle humanitaire” en débat en Europe et au Japon, 1890–1905’, *Le Mouvement Social*, 4 (2016), 93–108: 107.

71 ‘Les Balles humanitaires’, *L’Union médicale*, 56, 70 (1893), 829–36: 829.

72 ‘Théories émises sur la balle humanitaire; limite maxima de réduction du calibre des armes de guerre’, *Gazette des hôpitaux civils et militaires*, 1896, pp. 1313–14.

73 ‘Les Balles’, *op. cit.* (note 71), 835.

74 ‘Report on the experiments with various bullets against animals’, London, 1908, National Archives (Kew), WO 33/463.

75 La Garde, *op. cit.* (note 45), 114.

76
*Ibid.*

77 Stéphane Audoin-Rouzeau and Annette Becker, *14–18, retrouver la Guerre* (Paris: Éditions Gallimard, 2000), 51.

78 See Dirk Moses (ed.), Empire, Colony, Genocide: Conquest, Occupation, and Subaltern Resistance in the World History (New York: Berghahn Books, 2008).

79 It is to be noted that quickly, however, the question of the use of explosible bullets had been shadowed by the use of gas.

80

Mark Harrison , The Medical War: British Military Medicine in the First World War (Oxford: Oxford University Press, 2010), 27.

81 Anthony Bowlby, ‘The Bradshaw Lecture on Wounds in War’, *BMJ*, 2, 2869 (1915), 913–21: 913.

82 Médecin Chef Bonnat and Médecin traitant Duvillard, ‘Observation d’une blessure par balles explosive’, Archives diplomatiques (La Courneuve), 1 CPCOM 1098.

83
*Ibid.*

84 Telegram from the Ministry of foreign affairs, 11 September 1914, Archives diplomatiques (La Courneuve), 1 CPCOM 1099.

85 Capitan, ‘Présentation radiologique’, *Bulletin de l’Académie nationale de médecine*, 74 (1915), 51.

86 Lavielle, ‘Les Balles dum-dum austro-allemandes’, *Le Caducée. Colonie, guerre, marine*, 15, 12 (1915), 157–9.

87 Bourke, *op. cit.* (note 20), 137.

88 Section technique de l’artillerie, ‘Examen de projectiles employés par une des armées belligérante’, Paris, 20 August 1914, Archives diplomatiques (La Courneuve), 1 CPCOM 1098.

89 Audoin-Rouzeau and Becker, *op. cit.* (note 77), 143.

90 ‘Rapports et procès-verbaux d’enquête de la commission instituée en vue de constater les actes commis par l’ennemi en violation du droit des gens’, III–IV, 1916, 9.

91
*Ibid.,* 47.

92
*Ibid.*, 60.

93 Kaiser Wilhelm on the contemptible manner of waging war by England and France, National Archives (Kew), WO 32/4904.

94 Memorandum communicated by the War Office respecting British and German Service Ammunition, 7 October 1914, National Archives (Kew), WO 32/5085.

95 W.F. Stevenson, ‘On the Use of “Dum-Dum” and Explosive Bullets in War’, *BMJ*, 2, 2808 (1914), 701–2: 701.

96 ‘German Experience of Bullet Wounds’, *BMJ*, 2, 2810 (1914), 801.

97 K. Stargardt, ‘The English Infantry Bullets and Their Action’, *Journal of the Royal Army Medical Corps*, 24 (1915), 601–4: 601.

98
*Ibid.*, 604.

99 See M. Kirschener, ‘Remarks on the Action of the Regular Infantry Bullet and of the Dumdum Bullet on the Human Body’, *Journal of the Royal Army Medical Corps*, 24 (1915), 605–14; G. Perthes, ‘The Action of the Regular Infantry Bullet and the Dumdum Bullet on the Human Body’, *Journal of the Royal Army Medical Corps*, 25 (1915), 245–52.

100 Report on the Wounding Trials with Austrian Explosive Bullet, National Archives (Kew), WO 32/5556.

101 Eric Prokosch, *The Technology of Killing. A Military and Political History of Antipersonnel Weapons* (London and New Jersey: Zed Books, 1995), 13.

102 Paul Cornish, ‘Unlawful wounding: Codifying interaction between bullets and bodies’, in Paul Cornish and Nicholas Saunders (eds), *Bodies in Conflict: Corporeality, Materiality, and Transformation* (London: Routledge, 2014), 12.

103 Isabelle Montani, Eric G. Sapin and Christophe Champod, ‘Rodolphe Archibald Reiss, un criminaliste – criminologue au milieu de la guerre’, in Christophe Vuillemier (ed), *La Suisse et la guerre de 1914–1918* (Geneva: Slatkine, 2015), 545–575.

104 Rodolphe A. Reiss, ‘Les Balles explosibles autrichiennes’, *Archives de l’anthropologie criminelle*, 29 (1914), 897.

105 ‘Rapport succinct concernant les cartouches 1. 2. et 3. Remise par le Comité International de la Croix-Rouge, Geneva, 10 January 1916, Archives of the International Committee of the Red Cross (Geneva), A CS-053.

106

E. Newton Harvey et al., ‘Mechanism of Wounding’, in James C. Beyer (ed.), Wound Ballistics (Washington DC: Office of the Surgeon General Department of the Army, 1962), 152.

107
*Ibid.*, 143.

108 Draft report of the Commission on the Responsibilities for the War, 1919, 8, National Archives (Kew), FO 608/245/4.

109 Robin M. Coupland, ‘The Red Cross Classification of War Wounds: The E.X.C.F.V.M. Scoring System’, *World Journal of Surgery*, 16 (1992), 910.

